# Poster Session II - A317 PANCREATIC ADENOCARCINOMA DIAGNOSED ON STANDARD ESOPHAGOGASTRODUODENOSCOPY

**DOI:** 10.1093/jcag/gwaf042.317

**Published:** 2026-02-13

**Authors:** J Herblum, B Hersen, K Woodman

**Affiliations:** McGill University Faculty of Medicine and Health Sciences, Montreal, QC, Canada; Queen’s University Department of Medicine, Kingston, ON, Canada; McMaster University Division of Gastroenterology, Hamilton, ON, Canada

## Abstract

**Background:**

Pancreatic adenocarcinoma typically presents with abdominal pain or jaundice. Diagnosis is suspected with cholestatic liver enzyme elevation, cross-sectional imaging demonstrating a mass, and confirmed via endoscopic ultrasound with biopsy. GI bleeding as an initial presentation of pancreatic cancer is exceptionally rare; furthermore, direct visualization of a pancreatic mass on EGD has never been described to our knowledge.

**Aims:**

This study reports a case of newly identified pancreatic cancer invading into the duodenum, causing upper GI bleeding, in a patient not known to have a pancreatic malignancy. The risk factors for and mechanisms by which pancreatic cancer can cause upper GI bleeding were explored.

**Methods:**

A 67-year-old man with prostate cancer and suspected liver metastases presented with abdominal pain and developed an upper GI bleed following initiation of therapeutic anticoagulation for an acute pulmonary embolism. He required 3 units of packed red blood cells and was initiated on a pantoprazole infusion. Initial esophagogastroduodenoscopy (EGD) revealed a duodenal ulcer with an adherent clot. Second-look EGD revealed a deep, malignant-looking ulcer with a small blood clot. Abdominal imaging with a dedicated pancreas protocol and liver biopsies were consistent with metastatic pancreatic adenocarcinoma.

**Results:**

There are limited endoscopic studies conducted in patients with pancreatic cancer. In one study of 75 patients with pancreatic cancer, 53 underwent EGD at diagnosis; only 3 had gastroduodenal ulcers, and 11 had gastroduodenal invasion. Of the cohort, 46 patients had new upper GI lesions with pancreatic cancer progression. In a retrospective review, the median time from hemorrhage to mortality in patients with pancreatic cancer was 30 days. Risks for GI bleed with pancreatic cancer include initial cancer stage, tumour location in the pancreatic head, alcohol consumption, smoking status, and history of GI bleeding.

**Conclusions:**

There is a possible risk for misdiagnosis of invasive pancreatic cancer causing GI bleeding, and one should maintain a high level of vigilance when assessing malignant-appearing ulcers. Current guidelines recommend against routine second-look endoscopy, but it should be considered if adherent clot removal is not feasible.

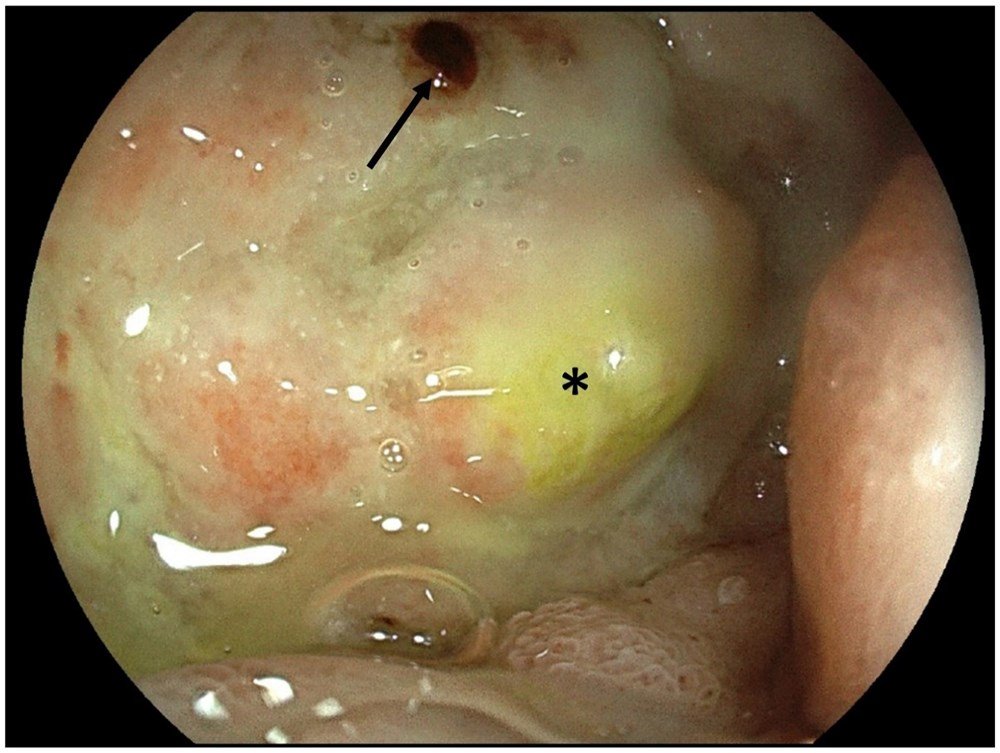

Second-look endoscopy with findings of a malignant appearing ulcer with small blood clot (arrow) and adjacent pancreatic mass invading the duodenal wall (*).

**Funding Agencies:**

Nones

